# Insight into the photodegradation mechanism of bisphenol-A by oxygen doped mesoporous carbon nitride under visible light irradiation and DFT calculations†

**DOI:** 10.1039/d2ra00995a

**Published:** 2022-04-06

**Authors:** Fatimah Bukola Shittu, Anwar Iqbal, Mohammad Norazmi Ahmad, Muhammad Rahimi Yusop, Mohamad Nasir Mohamad Ibrahim, Sumiyyah Sabar, Lee D. Wilson, Dede Heri Yuli Yanto

**Affiliations:** School of Chemical Sciences, Universiti Sains Malaysia Minden 11800 Penang Malaysia anwariqbal@usm.my; The Federal Polytechnic Offa P.M.B 420 Offa Kwara State Nigeria; Experimental and Theoretical Research Lab, Department of Chemistry, Kulliyyah of Science, International Islamic University Malaysia Bandar Indera Mahkota 25200 Kuantan Pahang Malaysia mnorazmi@iium.edu.my; Department of Chemical Sciences, Faculty of Science and Technology, Universiti Kebangsaan Malaysia 43600 Bangi Malaysia rahimi@ukm.edu.my; Chemical Sciences Programme, School of Distance Education, Universiti Sains Malaysia Minden 11800 Penang Malaysia; Department of Chemistry, University of Saskatchewan 110 Science Place, Room 165 Thorvaldson Building Saskatoon SK S7N 5C9 Canada; Research Center for Applied Microbiology, National Research and Innovation Agency (BRIN) Indonesia dede.heri.yuli.yanto@brin.go.id

## Abstract

Oxygen doped mesoporous carbon nitride (O-MCN) was successfully synthesized through one-step thermal polymerization of urea and glucose utilizing nanodisc silica (NDS) from rice husk ash as a hard template. The CO_2_ gas, NH_3_ and water vapor produced during the thermal process reshaped the morphology and textural properties of the of O-MCN compared to pristine mesoporous carbon nitride (MCN). Highest bisphenol A (BPA) removal achieved under visible light irradiation was 97%, with 60% mineralization ([BPA] = 10 mg L^−1^: catalyst dosage = 40 mg L^−1^; pH = 10; 180 min). In addition to mesoporosity, the sub-gap impurity states created from the oxygen doping reduced recombination rate of photogenerated carriers. Holes (h^+^) and superoxide (O_2_˙^−^) were identified as the predominant active species responsible for the photodegradation process. The photodegradation route was proposed based on the intermediates detected by LC-time-of-flight/mass spectrometry (LC/TOF-MS). The Density of States (DOS) showed that oxygen doping resulted in a higher photoactivity due to the stronger localization and delocalization of the highest occupied molecular orbital (HOMO) and lowest unoccupied molecular orbital (LUMO). The adsorption pathway of the BPA on the O-MCN and MCN was successfully predicted using the DFT calculations, namely molecular electrostatic potential (MEP), global and local descriptors.

## Abstract 2

Glucose is an excellent oxygen atoms donor for synthesising oxygen doped mesoporous carbon nitride (O-MCN). The oxygen doping created sub-gap impurity, preserved surface area and created broad pore size distribution to enhance the photodegradation of bisphenol A (BPA).

## Graphical abstract

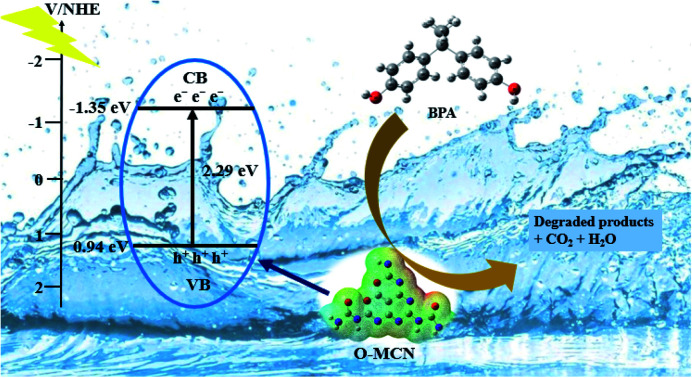

## Introduction

Bisphenol A (BPA), an endocrine-disrupting compound, is a common organic pollutant found in a variety of wastewaters around the world.^1^ Endocrine-disrupting chemicals (EDCs) can disrupt the functions of the endocrine systems in both animals and humans.^2,3^ Wastewater, sediments, surface waterways, groundwater, and even drinking water have all been shown to contain such compounds.^4^ In most cases, EDCs are difficult to decompose spontaneously into small organic molecules in actual conditions.^1^ Many approaches have been developed in recent years to remove BPA from water systems, including flocculation, adsorption, filtration, and other conventional wastewater treatment methods.^1,5^ Due to their poor and low adsorption capacity or high operating costs, these methods are confined to actual environmental remediation. As a result, finding a green, clean, and effective approach for degrading these refractory organic contaminants is extremely important. Photocatalytic degradation using photocatalysts are deemed to be the most potential approach in removing EDCs such as BPA from water bodies.

Carbon nitride (CN) is a semiconductor polymeric material that contains carbon and nitrogen in its framework. Graphitic carbon nitride (g-C_3_N_4_) is an analogue of CNs that has been widely investigated as a photocatalyst due to its narrow band gap (2.7 eV) and suitable redox potential. However, the recombination rate of electron/hole pairs in pure g-C_3_N_4_ is rapid.^6,7^ In addition to that, pure g-C_3_N_4_ has low electrical performance, restricted optical absorption, and low surface area.^8^ These characteristics caused the pure g-C_3_N_4_ to have poor photocatalytic activity.^9,10^ The pure g-C_3_N_4_ has been doped with metal-based and non-metal based dopants to overcome these limitations.^11–17^ Another strategy to improve the photocatalytic activity of pure g-C_3_N_4_ is by introducing mesoporosity.

Mesoporous carbon nitrides (MCNs) are one of the candidates that are widely considered in the photocatalytic degradation of BPA using various lights source since its discovery in 2005.^5^ The MCNs porous architecture increases the light harvesting properties and facilitates the mass transfer of guest species from the bulk to the surface of the photocatalysts in addition to suppressing the recombination rate of electron/hole pairs.^18,19^ Nevertheless, the MCNs low surface area, inert surface chemistry and poor conductivity hampers their full potential to be used in photocatalysis. Similar to g-C_3_N_4_, the MCNs have been doped with various metal-based and non-metal dopants to further improve its photocatalytic ability.

Liu *et al.*, recently reported that Fe atom clusters embedded N-doped graphene decorated with ultrathin mesoporous carbon nitride nanosheets (Fe-NG/MCNS) was able to remove 72% of BPA within 180 min compared to 17% by pristine MCN. However, the doping has resulted in the decrease of surface area from 107.77845 m^2^ g^−1^ to 2.9497 m^2^ g^−1^.^20^ Wang *et al.*, reported the photodegradation of BPA using mesoporous graphitic carbon nitride modified PbBiO_2_Br porous microspheres (mpg-C_3_N_4_/PbBiO_2_Br). The synthesized photocatalyst, resulted in the removal of 55% of BPA within 300 min. The N_2_ sorption analysis indicate the photocatalyst has lower surface area (49.61 m^2^ g ^−1^) compared to the pristine MCN (165.20 m^2^ g^−1^).^21^ Cyano-rich mesoporous carbon nitride nanospheres (MCNS) synthesized by Chen *et al.*, also showed good photodegradation of BPA. The MCNS achieved complete removal of BPA within 60 min. Unlike metal incorporated MCN, the BET surface area of the MCNS (30.55 m^2^ g^−1^) was higher compared to bulk g-C_3_N_4_ (3.93 m^2^ g^−1^).^22^ Sahu *et al.*, reported that mesoporous polymeric oxygen rich exfoliated graphitic carbon nitride (EGCN) successfully removed 99% of BPA with 120 min. Similar to MCNS, the EGCN has higher surface area compared to pristine g-C_3_N_4_.^23^ Even though, metal doping resulted in enhancement, the synthesis process is often time consuming and the possibility of the metal to leach into the reaction medium could lead to environmental concern. The reduction in the surface area after metal doping could potentially reduce the photodegradation efficiency of the photocatalysts especially during reusability. Considering these factors, metal-free MCNs are more appealing compared to metal doped MCNs.

Hence, the focus of this research is to synthesize oxygen doped MCN (O-MCN) *via* economical and environmentally friendly one-step thermal polymerization of urea, glucose and nanodisc silica from rice husk ash (hard template). The glucose was used as an oxygen donor. The synthesized O-MCN were subjected to several spectroscopic and microscopic analyses and their photocatalytic activities were assessed in the photodegradation of BPA under visible light irradiation. The effect of oxygen doping on the electronic properties of the photocatalysts (MCN and O-MCN) were calculated using the total density of states (TDOS) and partial density of states (pDOS) whereas the molecular electrostatic potential (MEP) was used to study the potential regions for nucleophilic and electrophilic attacks of the photocatalyst and BPA using a reactivity map. Global and local descriptors were carried out to validate and elucidate the reaction mechanism between the photocatalysts (O-MCN and MCN) and reactant (BPA).

## Experimental

### Materials

The chemicals used in this study were (>99%, QREC, Malaysia), glucose (>99%, Qrec, Malaysia), sodium hydroxide (>99%, Qrec), nitric acid (69%, Qrec, Malaysia), hydrofluoric acid (>49%, Qrec), bisphenol A (BPA, 97%, Acros Organics, Netherlands), ethylenediaminetetraacetate (EDTA, >98%, BDH Limited Poole England), ascorbic acid (>99.5%, Qrec) and methanol (AR grade, Qrec, Malaysia). All the chemicals were of analytical grade and used without further purification. Ultrapure water generated by Millipore Direct-Q™3 Ultrapure Water Purification System (Fisher Scientific, USA) was used to prepare the solutions. Rice husk was collected from a local rice milling company in Penang, Malaysia.

### Synthesis of nanodisc silica (NDS)

Rice husk ash (RHA) was used as a silica precursor in the synthesis of NDS according to the method reported by Bukola *et al.*^24^ Sodium silicate solution was prepared by dissolving the RHA (5.0 g) in 500 mL of aqueous NaOH solution (0.5 M) in a plastic container. Silica sol started to form when the pH of the sodium silicate was reduced to pH 7 using 2.0 M HNO_3_. The neutralized sodium silicate solution was vigorously stirred for 24 h followed by ageing in mother liquor for 48 h under a static condition for 48 h to form silica gel. After the ageing process, the silica gel was filtered and rinsed with distilled water. The freeze-drying technique was used to remove excess water from the NDS.

### Synthesis of mesoporous carbon nitride (MCN)

The MCN was prepared using the thermal polymerization method as reported by Xu *et al.*^25^ Ten grams of urea was used as the carbon and nitrogen source. The urea was dissolved in 50 mL of distilled water and heated to 70 °C under stirring. The NDS (0.2 g) was added to the urea solution as a hard template. The mixture was stirred at 80 °C until the water evaporated. The yellow MCN nanocomposite powder was calcined at 550 °C for 3 h at a heating rate of 5 °C min^−1^. The NDS was etched by dispersing the yellow powder in 100 mL of an aqueous solution of HF (5%) overnight. After 24 h, it was filtered using a filter paper, washed thoroughly with distilled water, and dried in the oven overnight at 80 °C. The yellow coloured MCN powder was kept in a desiccator for further use.

### Synthesis of oxygen doped mesoporous carbon nitride (O-MCN)

The O-MCN was synthesized similar to MCN with some modifications. Glucose (0.2 g) was added as a source of oxygen together with urea. The urea–glucose–NDS nanocomposite was calcined at 550 °C for 1 h instead of 3 h at a heating rate of 5 °C min^−1^. A dark, brown-coloured O-MCN was obtained and kept in a desiccator for further use.

### Characterization

The functional groups of the synthesized photocatalysts were identified using Fourier transform infrared (FT-IR) spectroscopy (PerkinElmer System 2000 FT-IR Spectrophotometer). The crystallinity of the photocatalysts was investigated using powder X-ray diffractions (XRD) (Siemens Diffractometer D5000 Kristalloflex, equipped with Cu Kα radiation, *k* = 0.154 nm) with a step size of 0.05° from 10° to 90°. The morphology of the samples was observed using scanning electron microscopy (SEM Leica Cambridge 360). The high-resolution transmission electron microscopy (HR-TEM) analysis was carried out by HRTEM 200 kV with Field Emission, TECNAI G2 20 S-TWIN, FEI, resolution below 100 nm. The nitrogen adsorption–desorption (NAD) analysis was done to determine the surface and porosity of the prepared photocatalysts. The solid-state UV/Vis diffuse reflectance spectroscopy (UV-Visible spectrophotometer – Cary 5000 UV NIS-NIR equipped with Lamda 35 software) was used to measure the bandgap of the prepared photocatalysts by scanning the reflectance from 200 to 800 nm. The photoluminescence analysis was done using RAMAN-PL Spectrometer, LabRam HR, Horiba with *λ*_max_ = 325 nm using laser power: 30 mW equipped with a helium–cadmium laser. X-ray spectrometer (XPS) is a quantitative surface technique (AXIS Ultra DLD, Kratos, equipped with a Al Kα X-ray source – 1486.6 eV at 10 mA and 15 kV to analyze a 300 μm × 700 μm area under 7.6 × 10^−9^ Torr ultra-high vacuum environment in the sample analyzing chamber) that was used in measuring the elemental. The XRD, FTIR, SEM and NAD analyses of NDS have been reported elsewhere.^24^

### Photocatalytic activity

The light source used for the photodegradation of BPA is made up of a 500 W xenon lamp (China) with a >400 nm cut-off filter glass (choot GG420 UV-filter). The cut-off filter was placed in between the solution and light source to prevent UV light from reaching the photocatalyst. The temperature of the reactor was regulated by air supplied by an aquarium aeration pump (model BB-8000) with an airflow rate of 20 mL min^−1^. The BPA solution (100 mL of 20 mg L^−1^) was stirred with a 50 mg L^−1^ photocatalyst under dark conditions for 30 min to achieve adsorption–desorption equilibrium. The solution was then irradiated with light for 180 min. At 30 min time intervals, 5 mL aliquots were sampled and filtered using 0.2 μm syringe filters to remove the photocatalyst particles and was further analysed by UV–Vis spectrometer (Schimadzu 2600 UV–Vis). This is to observe the changes in the absorbance in the range of 200–500 nm at room temperature using *λ*_max_ = 276 nm as reference.

### Computational chemistry

The Density Functional Theory (DFT) calculations were performed using the Gaussian09 program (Gaussian Inc., Wallingford, CT, USA). The three-dimensional (3D) structures of O-MCN, MCN and BPA (Chemspider ID: 6371) were drawn and taken from the Chemspider database, respectively. Conversely, due to the computational cost of studying MCN and O-MCN, the current study used only their monomers. The optimization of the structures was carried out in the gas phase at the 6-311++G(d,p) basis set level. The optimized geometry, HOMO, LUMO, and energy band gap were constructed using GaussView 5.0. The GaussSum program (version 3.0) was used to calculate the TDOS and pDOS of the catalyst above.^26^

### Statistical analysis

Four parameters were studied: pH, initial concentration of BPA, catalyst dosage, and the types of photocatalysts. Scavenging tests were conducted and expressed as mean ± standard deviation (SD). The photocatalytic activity (mean value) of the catalysts was validated by one-way analysis of variance (ANOVA) with Tukey's test using JMP pro 13.^27^ The effects of the parameters were established statistically significant at *p* < 0.05.

## Results and discussion

### Characterizations of the MCN and O-MCN

**Fig. 1:**
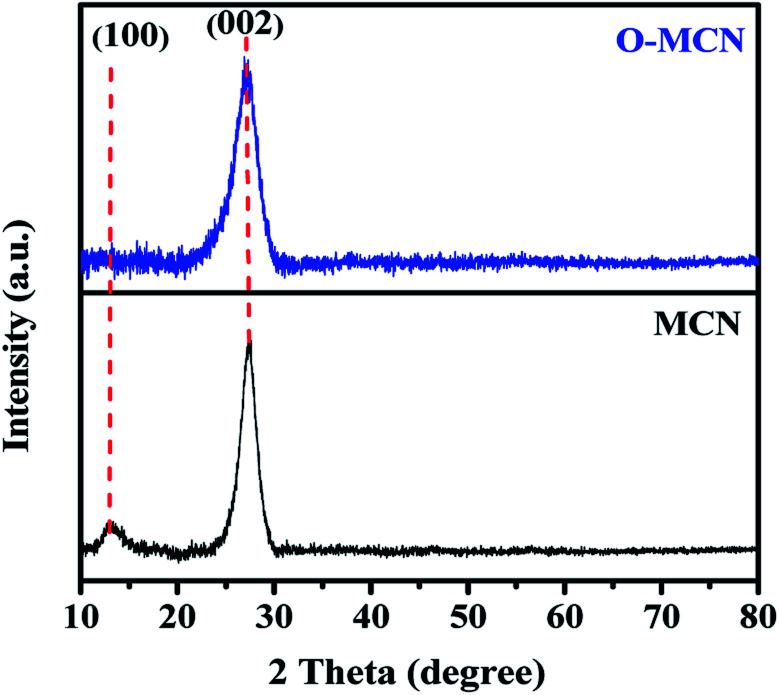
The XRD diffractogram of MCN and O-MCN.

The XRD pattern of MCN and O-MCN are presented in Fig. 1. The two peaks observed at 13.0° and 27.3° in the XRD diffractogram of MCN correspond to the in-planar structurally repeated motifs of triazine rings (indexed as (100)) and the interlayer reflection of the graphitic structure (indexed as (002)), respectively.^25^ The (100) peak disappeared, whereas the (200) peak becomes broader and slightly shifted to 27.1° when the MCN was doped with oxygen (O-MCN). The changes indicate that O-MCN has larger interlayer stacking distances and smaller crystallite sizes.^28,29^ The *d*-spacing values increased from 0.326 nm (MCN) to 0.329 nm (O-MCN), indicating the changes in the atomic structure of MCN due to oxygen doping.

The crystallite size of the photocatalysts was determined using the Scherrer formula according to eqn (1):^30^1
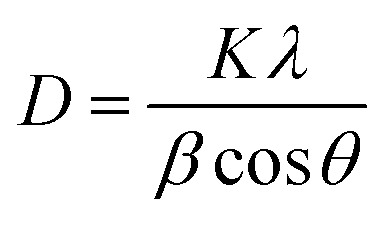
where *D* is the crystallite size (nm), *K* is the Scherrer constant (0.89), *λ* is the Cu-K_α_ radiation wavelength (0.15418 nm), and *β* is the full width of half maximum (FWHM) (002) diffraction peak at half maximum. The average crystallite size of O-MCN (3.21 nm) was lower than MCN (4.31 nm). The findings imply that the introduction of oxygen functionalities reduced the crystallinity of the MCN, causing the crystal plane spacing to widen, thus affecting the degree of polymerization. The XRD crystallographic parameters of the MCN and O-MCN are summarized in Table 1.

**Table 1:** XRD crystallographic parameters of MCN and O-MCN

Catalysts	2*θ*_(002)_ (°)	FWHM (°)	Crystallite size (nm)	Interplanar spacing (nm)
MCN	27.3	2.0931	4.31	0.326
O-MCN	27.1	2.7867	3.21	0.329

**Fig. 2:**
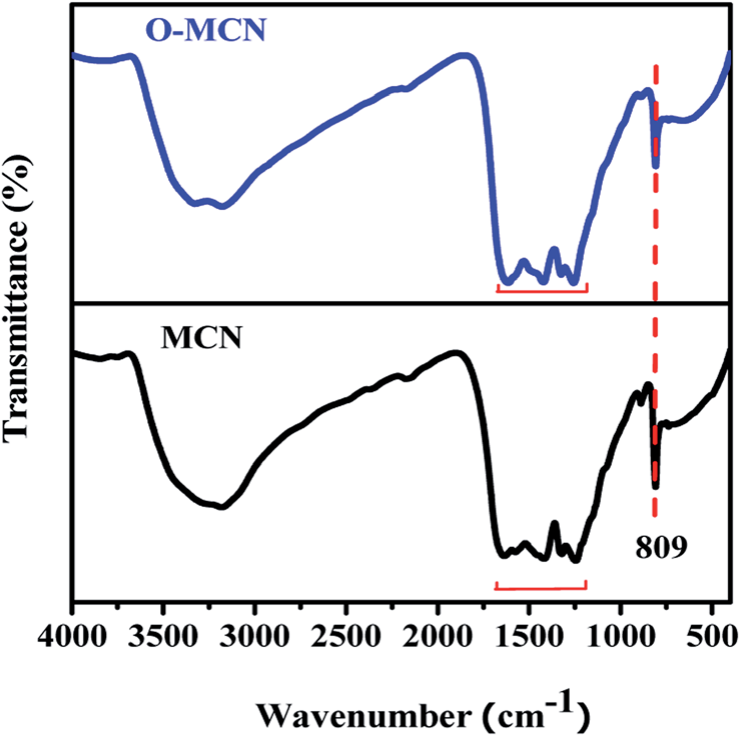
The FTIR spectra of MCN and O-MCN.

The FT-IR spectrum of MCN and O-MCN are shown in Fig. 2. The broad peak at 3000–3500 cm^−1^ represents the uncondensed NH and NH_2_ groups, which may overlap with the (H-bonded) OH stretch arising from adsorbed water. This peak appeared broader and less intense in the O-MCN spectrum. The change could result from a more complex environment for the O–H and N–H stretching vibrations on the edges of carbon nitride layers, implying more interlayer interaction.^31^ Reports in the literature indicate that for pure g-C_3_N_4_, the peaks in the range of 1200–1650 cm^−1^ are assigned to the trigonal (N–(C)_3_) and bridging C–NH–C units exemplifies the effective formation of the extended network of C–N–C bonds that make up the whole chemical nature of g-C_3_N_4_.^28^ The peaks are also referred to as the typical stretching vibration modes of C=N and C–N heterocycles.^32^ The intensity of these peaks was lower for MCN, and shifted slightly to lower wavelength for O-MCN. The shifting indicates the change in the C–N bond strength due to the presence of oxygen atoms. Even though changes were observed, the presence of these bands indicate that the heptazine structure is maintained.^33,34^ The sharp, intense peak observed at 809 cm^−1^ is assigned to the bending modes of triazine units. These are the building blocks for carbon nitride and were observed to decrease with the addition of glucose.^35^ Oxygen doping has irrefutably affected the C–N and N–H bonds, as evidenced by the transformation and shifting of these bands. These findings are in agreement with the XRD data, which confirmed the alterations in the triazine structure. However, since the C–O and C–N bonds appear in the same region, it is difficult to confirm the existence of C=O or C–O species in O-MCN nanocomposite using FT-IR.^29^ The XPS analysis was further carried out to investigate the oxygen environment in the O-MCN. The peak at 980 cm^−1^, which corresponds to the N–O groups, was not observed, indicating that the oxygen atom could be in the carbon nitride framework by substituting N atom in C–N=C bonds.^36^ The IR bands related to the siliceous framework were not observed in the FTIR spectrum of MCN and O-MCN indicating successful removal of silica during etching process.

**Fig. 3:**
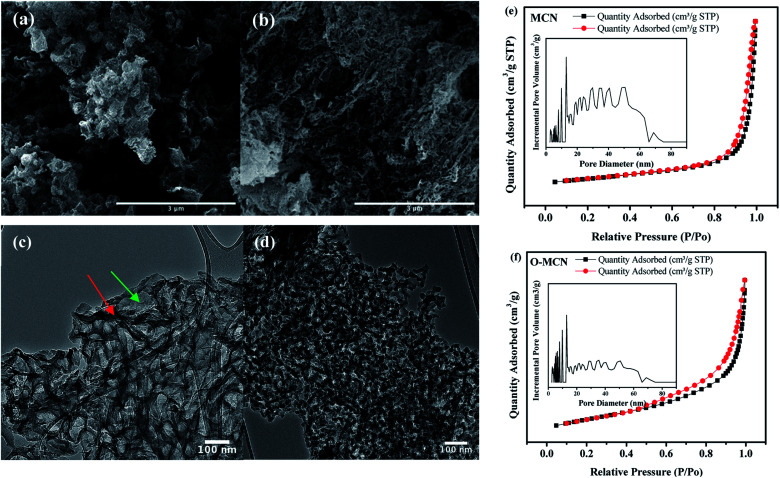
The SEM images of (a) MCN, (b) O-MCN and the TEM images of (c) MCN, (d) O-MCN. The N_2_ sorption isotherms (inset: NLDFT pore size distribution) are given in (e) and (f).

The microstructural characteristics and surface morphology of the MCN and O-MCN observed using SEM analysis are shown in Fig. 3. The cavities on the surface of MCN (Fig. 3(a)) are caused by the NH_3_ escaped in the form of gas during the polymerization process and etching of the NDS template. The SEM image of O-MCN shows that its sheet-like morphology is covered with cavities (Fig. 3(b)) as well. In addition to the release of NH_3_ gas and NDS etching, CO_2_ liberated from glucose decomposition, and water vapours could create cavities.^37^ In addition to creating cavities, the gases can potentially bind with the intermediates such as cyanuric acid, ammelide and melamine formed during the formation of O-MCN framework and interrupt the further development of the network structures.^38^

The TEM image of the MCN (Fig. 3(c)) indicates the presence of thin, overlapping, and crumbled nanosheets. After doping with oxygen, interconnected elongated rod-like nanoparticles with voids in between the particles could be seen (Fig. 3(d)). The changes indicate that besides being an oxygen source, glucose played a role in shaping the O-MCN nanocomposite. The CO_2_ gas produced from the glucose decomposition may have ruptured the thinner parts (shown in red arrows) of the nanosheet when escaping to the environment, leaving behind thicker parts (shown in green arrows).

The NAD analysis was used to determine the Brunauer–Emmett–Teller (BET) surface area and the pore structure of the MCN and O-MCN. The presence of type IV isotherm with an H3 hysteresis loop can be observed for all samples in the adsorption–desorption isotherms shown in Fig. 3(e) and (f). According to the International Union of Pure and Applied Chemistry (IUPAC) classification, the type IV isotherm indicates the presence of mesopores.^39^ The hysteresis loop of type H3 indicates ‘slit-shaped pores’ development from aggregates of plate-like particles.^40^ The BET surface area of the MCN and O-MCN was calculated to be 101 and 145 m^2^ g^−1^, respectively. The pore volume of O-MCN (0.19 cm^3^ g^−1^) was also obtained to be lower than MCN (0.29 cm^3^ g^−1^) possibly due to the structural collapse during etching and heat treatment.

The precise measurement of pore size distribution was obtained from the Non-Local Density Functional Theory (NLDFT) and the results are shown in the inset of Fig. 3(e) and (f). The wider pore size distribution of O-MCN compared to MCN is attributed to the liberation of excess CO_2_ and NH_3_ gasses in addition to water vapor. The textural changes may have also caused the changes in the pore size distribution.

**Fig. 4:**
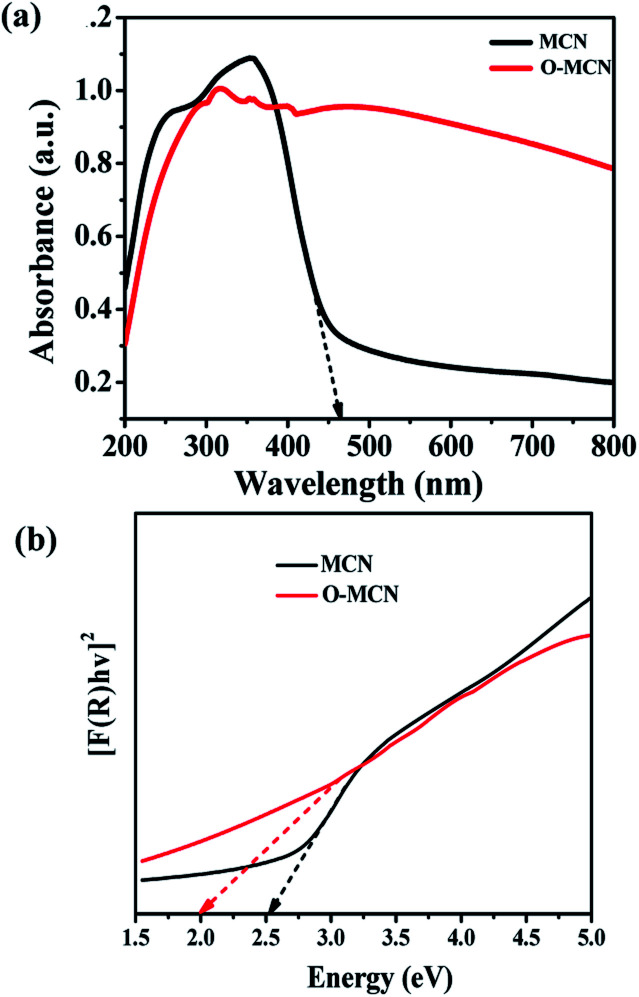
The (a) UV-Vis absorption spectra and (b) Kubelka–Munk plot of MCN and O-MCN.

The UV-vis DRS spectrum of MCN (Fig. 4(a)) shows that the MCN have better absorption in the UV region (200–400 nm) compared to the visible region (400–700 nm). The addition of oxygen enhanced the light absorption of O-MCN from UV to the visible range. The Urbach's tail which can be observed in the spectrum of MCN, could not be seen clearly in the spectrum of O-MCN. This phenomenon is known as sub-gap absorption. The Urbach's tail was observed to be increased in the absorption spectrum of O-MCN. The addition of oxygen reduced the amount of energy required to induce the sub-gap excitation.

In Fig. 4(b), the band gap energies (*E*_g_) of the MCN and O-MCN were determined and compared using the Kubelka–Munk method.^40^ A Kubelka–Munk plot can be used to evaluate the nanomaterials band gap where (*F*(*R*) × *hν*)^1/2^ was plotted against photon energy, *hν*. Kubelka–Munk function, *F*(*R*) is defined according to eqn (2):2
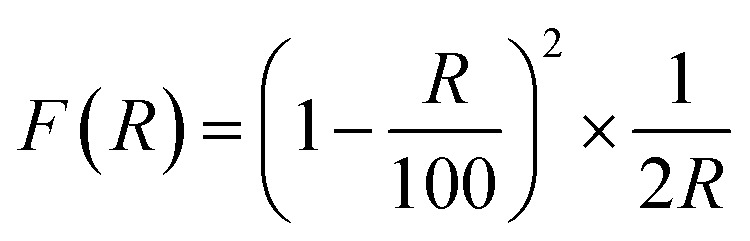
where *R* is the diffuse reflectance.

In the meantime, the reported wavelength, *λ* was used in calculating the *hv* and band gap energy, using eqn (3)3
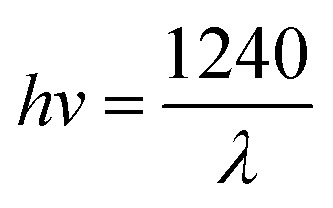


For MCN and O-MCN, the corresponding band gaps were calculated to be 2.50 and 2.29 eV, respectively. In principle when the size of a crystallite became smaller, the band gap energy will increase due to quantum confinement.^41^ However, the opposite was observed here. The observation was probably caused by the difference in morphology and crystallinity. Based on the UV-vis DRS analysis, it can be predicted that O-MCN will have better photocatalytic activity compared to MCN.

**Fig. 5:**
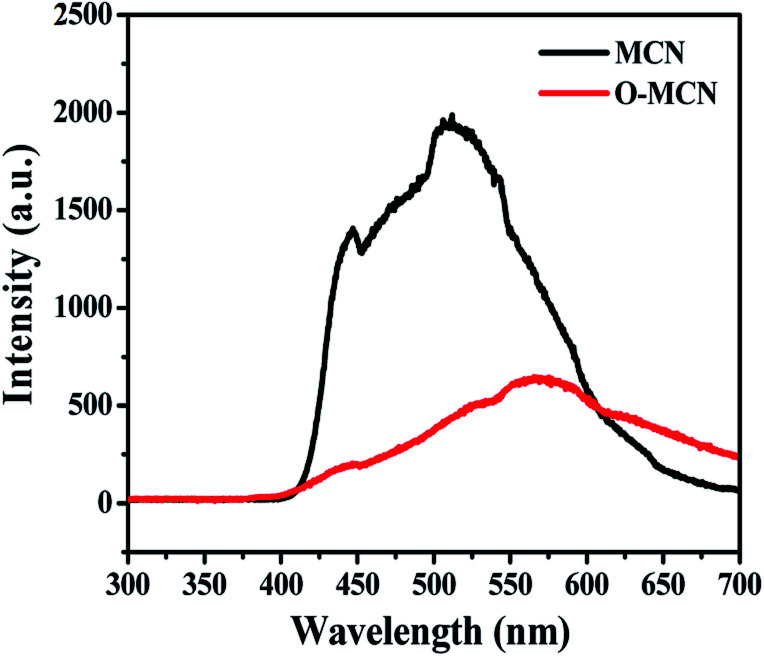
The PL spectra of MCN and O-MCN.

Fig. 5 represents the PL spectra of MCN and O-MCN excited by 325 nm at room temperature. The PL intensity of O-MCN is lower compared to MCN, indicating lower recombination rate of photogenerated electron–hole pairs. The defects created by the bound O atoms on the tri-*s*-triazine structure have effectively suppressed the photogenerated charge carriers from recombining.^42^ The defects could have acted as a centre to trap photoinduced electrons.^43^ The red-shifting of the O-MCN's PL maximum is linked to the reduced bandgap energy of the O-MCN. The inhibited recombination of band-to-band electron–hole and faster transfer of interfacial charge would increase the photogenerated charge carriers' separation efficiency.^44,45^

**Fig. 6:**
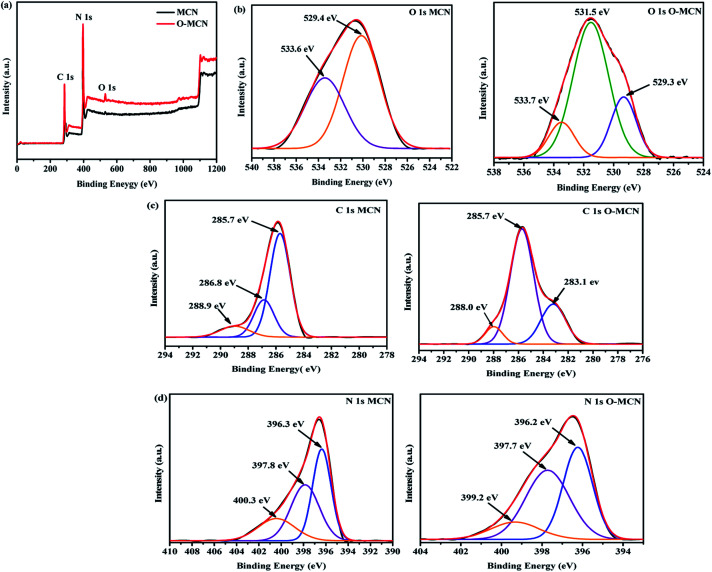
The XPS (a) survey scans, (b) C 1s, (c) N 1s and (d) O 1s narrow scans for MCN and O-MCN.

The X-ray photoelectron spectroscopy (XPS) measurement was conducted to obtain an insight into the structural binding of the elements present in the synthesized photocatalysts. The XPS survey spectra (Fig. 6(a)) clearly show that only C, N, and O elements are present in MCN and O-MCN. The composition of the elements is shown in Table 2.

**Table 2:** XPS elemental compositions of MCN and O-MCN

Samples	C (wt%)	N (wt%)	O (wt%)	C/N molar ratio
MCN	30.67	67.56	1.77	0.454
O-MCN	34.79	61.28	3.93	0.568

A prominent peak observed at 530 eV in the survey scan spectrum of O-MCN indicates the presence of oxygen within its framework. Deconvolution of the O 1s (Fig. 6(b)), peak resulted in three peaks at 529.3, 531.5, and 533.7 eV. The peak that appeared at 531.5 eV is attributed to the C–O, C=O, and N–C–O components in the O-MCN framework.^46,47^ The peaks at 529.3 and 533.7 eV are assigned to the surface hydroxyl and irreversibly adsorbed molecular water.^48^ These peaks are also observed in the narrow scan of MCN. Based on the O 1s peak analysis, it can be inferred that O atoms have replaced the N atoms.

The C 1s (Fig. 6(c)) of MCN is fitted with three peaks at 285.7, 286.8, and 288.9 eV. These peaks correspond to the N–C=N,^43^ C–NH_2_,^49^ and C–N–C^47^ components of the triazine ring. As for O-MCN, the peak at 283.1 eV attributed to C=O.^50^ The peak at 285.7 eV obtained in the O-MCN spectrum could also be assigned to C–O and N–C=N components. The peak observed at 288.0 eV is attributed to corresponds to the sp^2^-hybridized C (N–C=N) in the aromatic skeleton rings.^47^

Deconvolution of N 1s peak (Fig. 6(d)), of MCN and O-MCN resulted in three peaks centred at 396.3, 397.8, and 400.0 eV assigned to sp^2^ nitrogen (C–N–C), sp^3^ nitrogen, and amino functional groups with a hydrogen atom (C–NH) or C=N.^51^ The reduction in nitrogen content further proves that the O atoms have substituted the N atoms in the MCN framework.

### Photodegradation of bisphenol A

The BPA molecule can exist as an anionic (p*K*_a1_) or dianionic (p*K*_a2_) species under acid or alkaline conditions.^52^ Therefore, the effect of initial pH on the efficiency of BPA photodegradation was investigated in the range of 3 to 11. The BPA removal profile at different initial pH is shown in Fig. S1(a).† Under the dark conditions for 30 min, it was observed that about 94% of the BPA was significantly (*p* < 0.05) adsorbed in the pH range 3–9, whereas at pH 10 and 11, the adsorption was 54% and 27%, respectively. When irradiation started, significant photodegradation was not observed at pH 3–9 whereas the removal percentage at pH 10 and 11 increased to 91% and 80%, respectively. The BPA molecule dissociates and exists in aqueous system as mono BPA^−^ or divalent BPA^2−^ anions within its p*K*_a1_ (9.59) and p*K*_a2_ (11.30). At pH 10 and 11, the surface of the O-MCN will be negatively charged since its isoelectric point (pH_PZC_) was 4.8. The adsorption was found to be lower at this pH range due to stronger electrostatic repulsion between the formed bisphenolate anions (BPA^−^ and BPA^2−^) and the negatively charged surface of O-MCN.^53^ Since, the surface of the O-MCN is not saturated with the BPA, light can reach its surface and generate more reactive oxygen species (ROS) for the photodegradation process. The high adsorption observed at pH 3–9 could be due to other interactions such as chemical bonding between the BPA and the free site active groups such as NH_2_ and –OH in addition to stronger electrostatic attractions.^54^ Due to the highest photocatalytic removal of BPA at pH 10, the subsequent investigations were carried at pH 10.

The photocatalytic performance of O-MCN was evaluated by the Langmuir–Hinshelwood model as shown in eqn (4):4
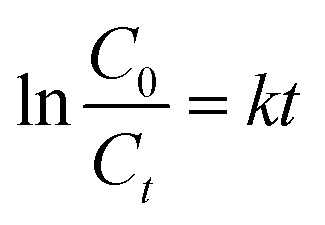
where *C*_0_ is the concentration of BPA prior to photocatalysis (mg L^−1^), *C*_*t*_ is the concentration of BPA (mg L^−1^) at photocatalytic reaction time *t* (mg L^−1^), and *k* is the photocatalytic reaction rate constant (mg L^−1^). The *k* values are calculated from the slope of −ln(*C*_*t*_/*C*_0_) *versus t* plot. Fig. S1(b)† displays the pseudo-first-order kinetics for BPA removal using O-MCN at different pH media, under visible light irradiation within 180 min. The calculated *k* values at pH 10 and 11 are 0.0133 and 0.0060 min^−1^, respectively, reflecting the maximum rate constant at pH 10 for BPA degradation.

The effect of the initial concentration of BPA on the photodegradation is shown in Fig. S2(a).† It was observed that the adsorption of BPA was higher at an initial concentration of 5 and 10 mg L^−1^ due to the higher concentration of available adsorption sites on the O-MCN compared to the pollutant molecules. As the initial concentration was increased to 20–50 mg L^−1^, the amount of adsorbed BPA reduced. This trend is expected since more active sites have been occupied by the BPA molecules. Surprisingly, regardless of adsorption level, the final removal of the BPA was determined to be in the range of 80–90%. This trend is possibly due to the ability of O-MCN to generate generous amount of highly energetic reactive oxygen species (ROS) in a short period of exposure to light to breakdown the BPA molecules.

The photodegradation of BPA at different initial concentrations was determined to follow the pseudo-first-order kinetics. The calculated *k* values for initial concentration of BPA at 5, 10, 20, 30, 40 and 50 mg L^−1^ were 0.0074, 0.0081, 0.0077, 0.0073, 0.0067 and 0.0052 min^−1^, respectively. Even though the final removal of BPA is almost the same, based on the *k* values, the speed of BPA removal varies significantly. As shown in Fig. S2(b),† within 180 min of light irradiation, 90% photodegradation of was observed for 10 mg L^−1^ BPA by O-MCN. Due to its fastest removal rate, it was chosen to be used in the optimization of other parameters.

The effect of photocatalyst dosage on the photodegradation of BPA is shown in Fig. S3(a).† From the figure, 40 mg L^−1^ photocatalyst dose appeared to be optimal, yielding 97% of BPA removal. Further increase in the dosage resulted in slight decrease in the removal percentage. The excess amount of O-MCN in the BPA solution may agglomerate and reduce its available surface area for photocatalytic reaction. This condition may also prevent the particles from receiving the light for ROS generation.^55^ The effect of photodegrading BPA is therefore minimized.

The photodegradation of BPA at different photocatalyst dosages follows the pseudo-first-order kinetics as shown in Fig. S3(b).† The reaction rate constant of the photodegradation BPA is 0.0063, 0.0133, 0.0081, 0.0059 and 0.0092 min^−1^, respectively. The effect of O-MCN dosage significantly (*p* < 0.05) improved BPA photodegradation as the rate constant (*k*) changed from 0.0062 to 0.0133 min^−1^.

The BPA removal efficiency catalysed by O-MCN was compared to photolysis, P25 and MCN. As shown in Fig. S4(a),† the O-MCN exhibited highest photocatalytic activity compared to P25, and MCN. The BPA photodegradation efficiency reached significantly (*p* < 0.05) 97% for O-MCN, 45% for P25, and 84% for MCN. The BPA was not photodegraded without the presence of any photocatalysts. The plots of −ln(*C*_*t*_/C_0_) *versus t* indicate that photocatalytic BPA degradation fits well by pseudo-first-order kinetic with good correlation coefficients as shown in Fig. S2(b).† The measured *k* values for photolysis, P25, MCN and O-MCN are 0.0015, 0.0046, 0.0105 and 0.0132 min^−1^, respectively.

Based on the collected physicochemical and photocatalytic analyses, the superior photocatalytic ability of O-MCN is attributed to doping of oxygen atoms and highly porous nature of the O-MCN. The higher porosity of O-MCN and sub-gap impurity states produced by the oxygen atoms in its electronic band structure enhanced light-harvesting capability. The oxygen dopant also effectively captures the electrons and mediated the interfacial charge transfer to enhance photocatalytic efficiency.^56^

**Fig. 7:**
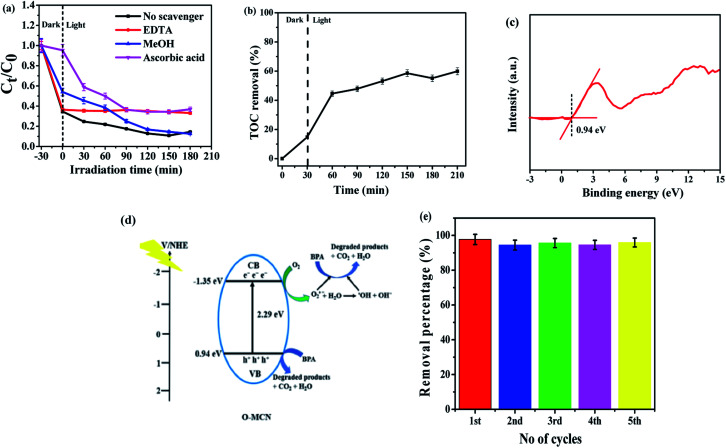
The (a) effect of active species scavengers and (b) the TOC result on the photodegradation efficiency of BPA using O-MCN. The (c) VB-XPS spectra and (d) a schematic illustration of the proposed mechanism for the photodegradation of BPA using O-MCN photocatalyst. (e) The reusability studies of O-MCN on the photodegradation efficiency of BPA.

Scavenging test was performed to identify the ROS that is responsible for the photocatalytic degradation activities such as the positively charged holes (h^+^), hydroxyl radicals (˙OH) and superoxide radicals (O_2_˙^−^). The O_2_˙^−^, h^+^, and ˙OH scavengers used were ascorbic acid (AA), ethylenediaminetetraacetic acid (EDTA) and methanol (MeOH), respectively. Fig. 7(a) illustrates the effect of chemical scavengers on the photocatalytic activity of O-MCN. The photodegradation efficiency of BPA was inhibited when EDTA was added, suggesting that the h^+^ played an essential role in the photodegradation of BPA using O-MCN. With the addition of AA, the rate of photodegradation of BPA also decreased, suggesting that O_2_˙^−^ also plays a crucial role in photocatalytic degradation. However, no major decrease is seen in the photocatalytic degradation rate of BPA in the presence of MeOH, suggesting that the contribution of ˙OH is minimum. It can therefore be inferred that the h^+^, O_2_˙^−^ and ˙OH radicals took part in the photodegradation of BPA with O-MCN under visible light irradiation, with significant contributions from h^+^ and O_2_˙^−^ rather than ˙OH.

The mineralization effect of the O-MCN on BPA was investigated using TOC analysis. The first 30 min of irradiation resulted in about 15% TOC removal through adsorption in the dark as shown in Fig. 7(b). After 180 min of irradiation, about 60% TOC removal was achieved. The result shows that the O-MCN could effectively mineralize BPA under visible light irradiation.

The band alignment structure of O-MCN was proposed based on the UV-vis DRS and Valence Band X-ray Photoelectron Spectroscopy (VB-XPS) analyses. The band edge position for O-MCN from absolute electronegativity was determined using eqn (5) and (6):

5 *E*_VB_ = *X* − *E*^e^ + 0.5*E*_g_

6 *E*_CB_ = *E*_VB_ − *E*_g_

where *E*_VB_ is the edge potential of the valence band (VB), *E*_CB_ is the edge potential of the conduction band (CB), *E*_g_ (2.29 eV) is the semiconductor bandgap energy, *X* is the semiconductor electronegativity that is the geometric mean of the constituent atoms' electronegativity and on the hydrogen scale (∼4.5 eV), *E*^e^ is the energy of free electrons. The VB-XPS of O-MCN is 0.94 eV as shown in Fig. 7(c). The O-MCN CB edges *vs.* NHE are determined to be – 1.35 eV. The CB potentials O-MCN is more negative than *E*^0^(O_2_/O_2_˙^−^) = −0.33 V *vs.* NHE, indicating that the electrons in the reaction will react with O_2_ to produce O_2_˙^−^. The VB potential of O-MCN is lower than *E*^0^(˙OH/OH^−^) = +1.99 V *vs.* NHE. As a result, photogenerated holes are unable to oxidize OH^−^ into ˙OH radicals.

During irradiation, the photogenerated electrons migrated to CB leaving behind photogenerated h^+^ at the CB. The holes in the VB can react directly with BPA, while the photogenerated electrons can react with oxygen to produce O_2_˙^−^ radicals. The photodegradation process by the ROS is simplified in eqn (7)–(13).

7 O-MCN + *hv* → O-MCN (h_VB_^+^ + e_CB_^−^)

8 O-MCN–h^+^ + BPA → intermediate + CO_2_ + H_2_O

9 O-MCN–e^−^ + O_2_ → O_2_˙^−^

10 2O_2_˙^−^ + 2H_2_O → H_2_O_2_ + 2OH^−^ + O_2_

11 H_2_O_2_ + e^−^ → ˙OH + OH^−^

12 O_2_˙^−^ + BPA → intermediate + CO_2_ + H_2_O

13 ˙OH + BPA → products

A photodegradation mechanism was proposed based on the collected data and findings and the previous report.^57–59^ The schematic diagram is shown in Fig. 7(d).

Fig. 7(e) shows the reusability of O-MCN up to five cycles. The photocatalyst is extremely stable under visible light irradiation for BPA degradation and can be used for long-term water treatment. The O-MCN's high durability and reusability rate provide good justification for the economic and practical use of the photocatalytic treatment system.

The LC/TOF/MS analysis detected several intermediate products formed during the photodegradation process. The photodegradation of BPA is proposed to take place through two different ways; hydroxylation reaction by O_2_˙^−^ with the generation of monohydroxylated BPA (243 *m*/*z*), and the β-scission with the formation of isopropenylphenol (*m*/*z* = 134) and phenol (*m*/*z* = 94) after it was attacked by photogenerated h^+^ and O_2_˙^−^. The earlier formed monohydroxylated BPA can continue to undergo hydroxylation to form dihydroxylated BPA (*m*/*z* = 259) and to 4-isopropenylphenol (*m*/*z* = 134) through hydroxylation.^60^

**Scheme 1:**
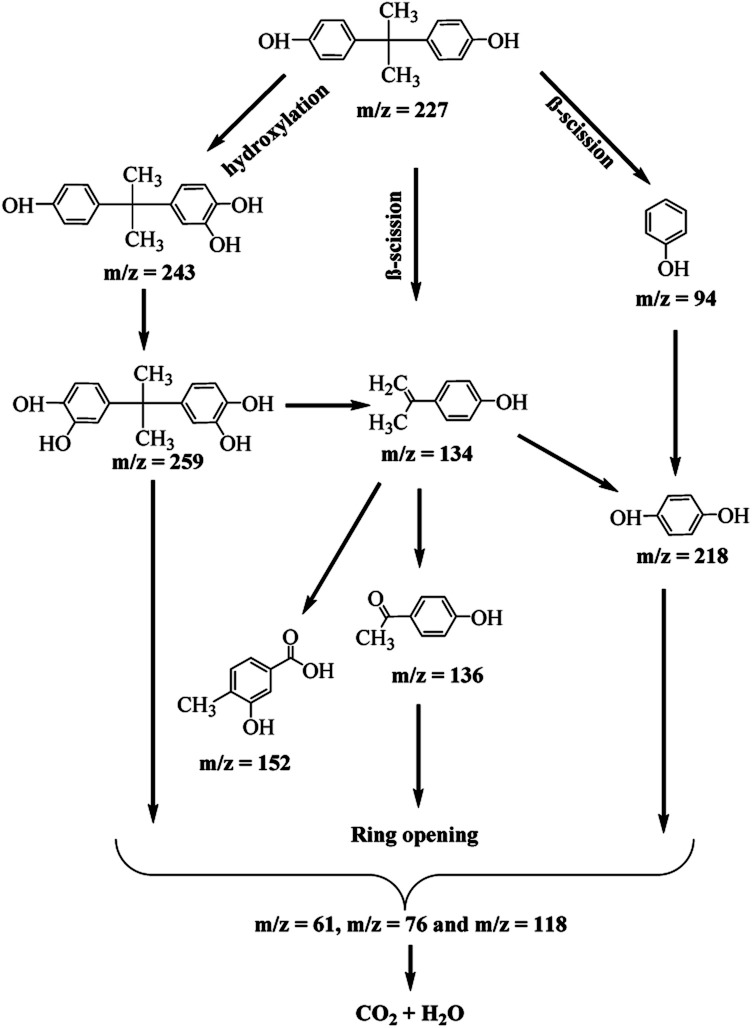
The proposed possible pathways during the photocatalytic degradation of BPA in the presence of O-MCN.

In a similar reaction, the phenol and 4-isopropenylphenol were further degraded to decomposable hydroquinone (*m*/*z* = 218) through hydroxylation and 4-hydroxyacetophenone (*m*/*z* = 136) by chemical oxidation reaction.^61,62^ The oxidation of 4-isopropylphenol will yield 4-hydroxyacetophenone and 3-hydroxy-4-methoxy benzoic acid (*m*/*z* = 152).^63,64^ It is proposed that at the end of the photodegradation process, the aromatic products created were broken down into simple organic acids such as maleic acid, glycolic acid and acetic acid which were further converted into CO_2_ and H_2_O through the combined oxidation of photogenerated h^+^ and O_2_˙^−^. During the ring-opening of aromatic compounds, hydroquinone was commonly considered to be a significant intermediate.^61^ The potential photodegradation pathway for BPA over O-MCN is simplified in Scheme 1.

### Computational chemistry

**Fig. 8:**
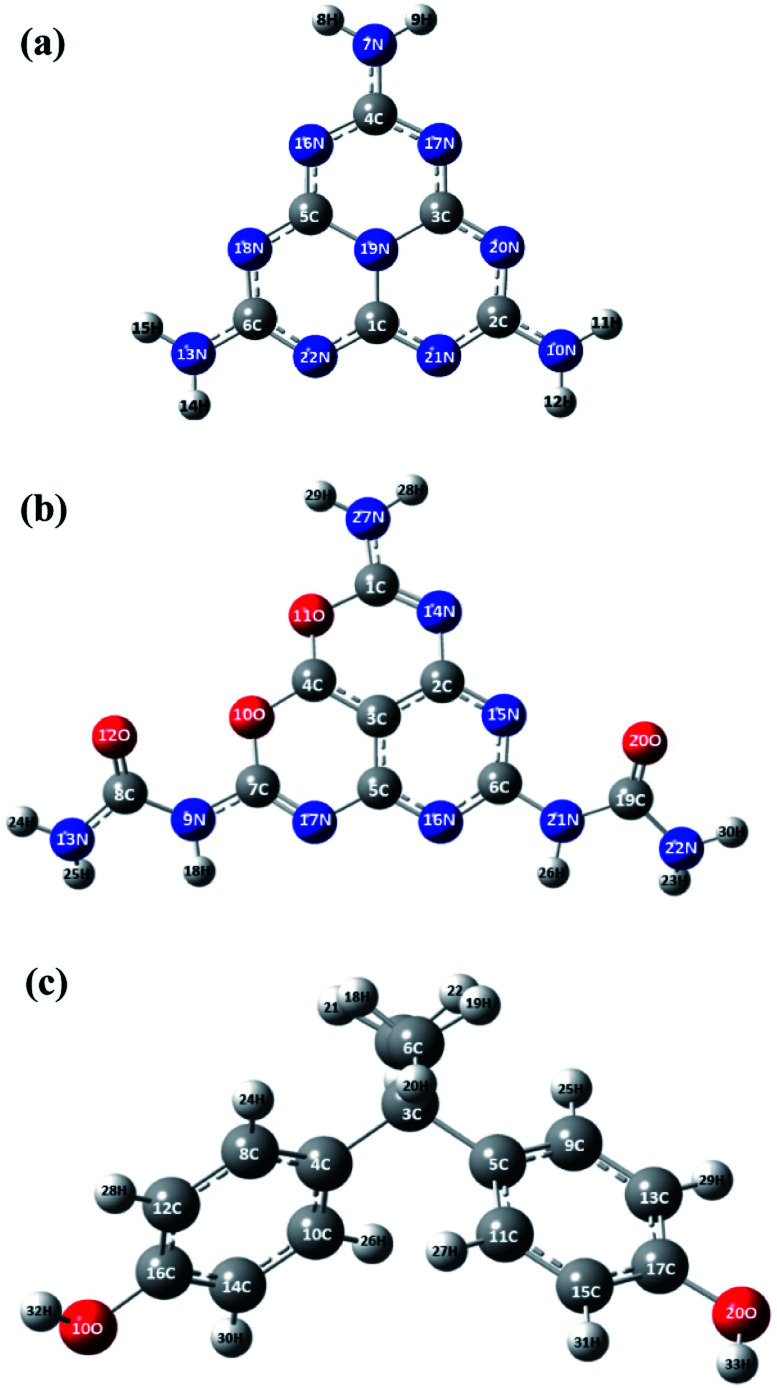
The optimized structure of (a) MCN, (b) OMCN and (c) BPA.

**Fig. 9:**
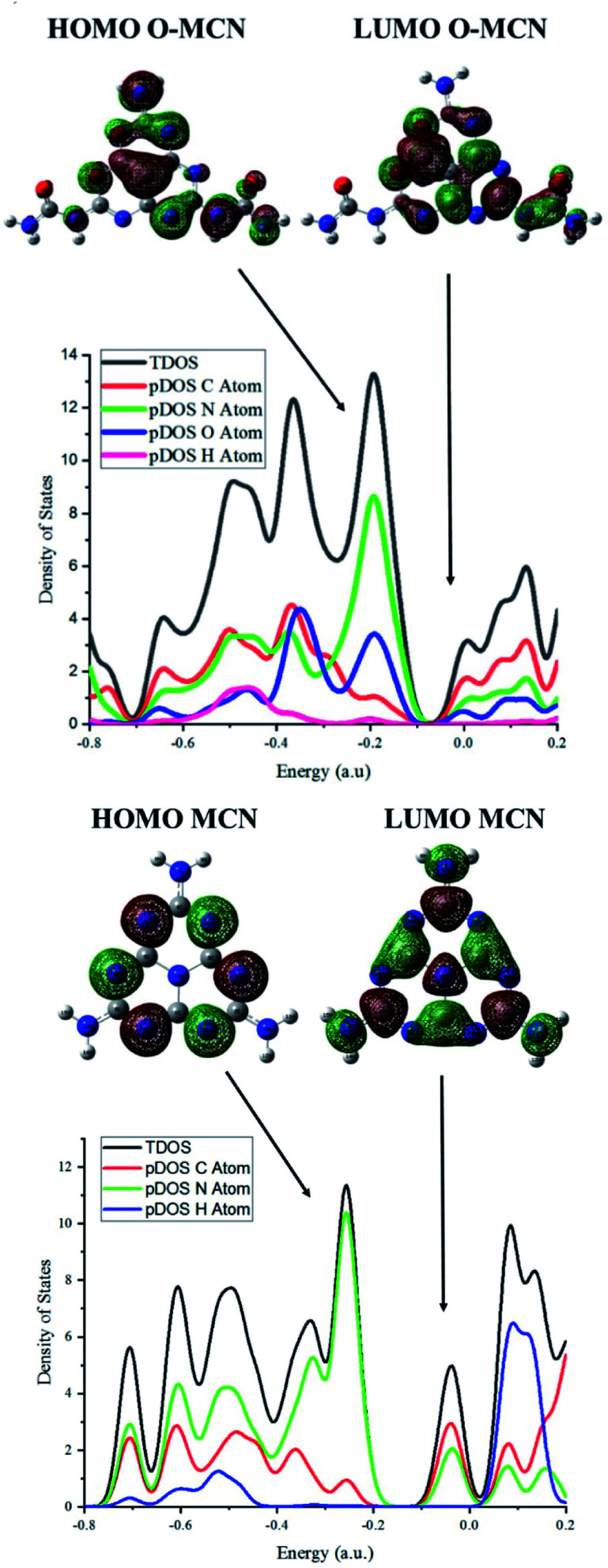
The DOS and pDOS of the O-MCN and MCN.

To further investigate the effect of oxygen doping, the electronic properties of the MCN and O-MCN were calculated using the TDOS and pDOS. The TDOS and pDOS are effective methods for visually characterizing the frontier molecular orbital (FMO) compositions.^65^Fig. 8 and 9 show the optimized structures of MCN, O-MCN and BPA and DOS of the MCN and O-MCN, respectively. Fig. 8 indicates that oxygen contributed significantly to the HOMO–LUMO gap, where O-MCN has a lower HOMO–LUMO gap than MCN.

This phenomenon explains the higher photoactivity of O-MCN compared to MCN. Notably, the pDOS of MCN suggests that the LUMO and HOMO are comprised mostly of carbon and nitrogen, and nitrogen, respectively. In contrast, the pDOS of O-MCN shows that the LUMO and HOMO are comprised mostly of carbon and nitrogen, and nitrogen and oxygen, respectively. In addition, oxygen doping resulted in the stronger localization and delocalization of the HOMO and LUMO in O-MCN than in MCN. The enhancement of the redistribution of the HOMO and LUMO is favourable for improving the carrier mobility, and consequently, the photocatalytic activity has been explained in detail in the experimental results above.

**Fig. 10:**
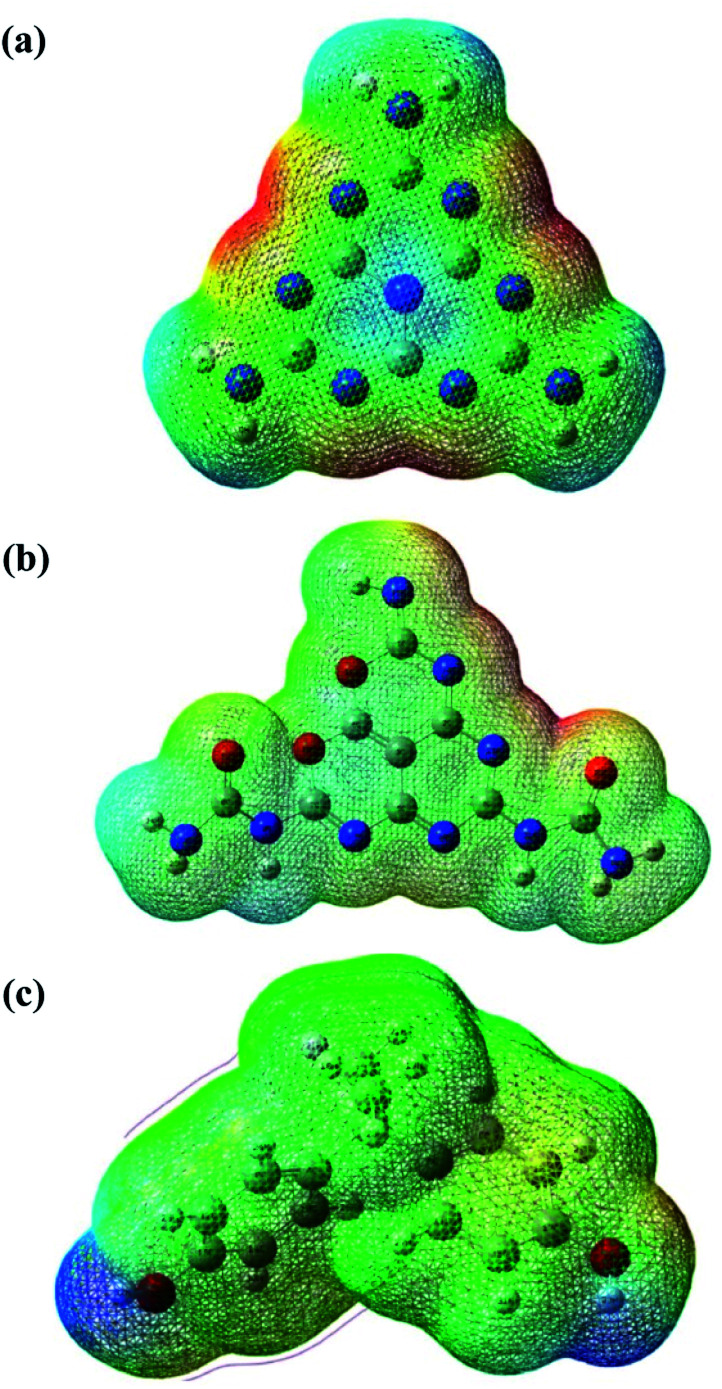
The MEP of (a) MCN, (b) O-MCN and (c) BPA structure.

The molecular electrostatic potential (MEP) is a theoretical approach that has been widely used in catalyst science to study potential regions for nucleophilic and electrophilic attacks of the photocatalysts and BPA using a reactivity map.^66^ The regions of this surface potential have been classified and ranked into five different colours, in the following order, red > orange > yellow > green > blue. As can be seen in Fig. 10, the deep red and blue regions surrounding the N (7, 10, 13, 16, 17, 18, 19, 20, 21), N (14, 15, and 27), and O (1 and 2) atoms represent the highest probability region for an electrophilic attack. In contrast, the blue region at the H (8, 9, 11, 12, 14, and 15), H (23, 24, 25, and 30), H (32 and 33) atoms represents the likely nucleophilic attack regions during the reaction between the photocatalysts and reactant (refer to Fig. 8 for atom numbering). To better understand the reaction mechanism between the photocatalysts (O-MCN and MCN) and the BPA, global and local descriptors were determined in the next section.

The global reactivity descriptors of MCN and O-MCN and BPA were calculated and evaluated to study the relationship between the stability, chemical reactivity, and structure. The HOMO–LUMO gap is one of the best predictors of the non-covalent interaction between the photocatalysts and the reactant.^67^ The energy of the HOMO–LUMO gap is one important parameter to compare the reactivity of the O-MCN and MCN toward BPA. The lower the gap, the higher the reactivity of the photocatalysts.

Based on the HOMO–LUMO gaps in Table 3, the reactivity of the photocatalysts and BPA can be arranged in the ascending order; MCN (4.6613 eV) < BPA (3.9456 eV) < O-MCN (0.3.460 eV). Based on the values, during the photodegradation of BPA using MCN as photocatalyst, the MCN and BPA acts as nucleophile and electrophile, respectively. Whereas, when O-MCN was used, it acts as a nucleophile and BPA acts as an electrophile.

**Table 3:** DFT calculation of quantum chemical parameters of BPA, MCN, and O-MCN

Name	*E*_HOMO_ (eV)	*E*_LUMO_ (eV)	*η* (eV)	*μ* (eV)	*ω* (eV)	*χ* (eV)	*σ* (eV)	*E*_LUMO_ − *E*_HOMO_ (eV)	Δ*N* (eV)
BPA	−8.6363	−4.6907	1.9728	−6.6635	0.0592	6.6635	375.3283	3.9456	1.9728
MCN	−8.1819	−3.5206	2.3306	−5.8512	0.0539	5.8512	317.7034	4.6613	2.3306
O-MCN	−3.6654	−0.2294	1.7180	−1.9474	0.0044	1.9474	431.0018	3.4360	1.7180

The chemical stability and reactivity of the compounds were also calculated using the absolute hardness (*η*) and softness (*σ*). The lower the value of *η*, the higher the reactivity of the compounds. In this study, MCN (2.3306 eV) has a higher *η* than BPA (1.9728 eV) and O-MCN (1.7180 eV), whereas O-MCN (431.0018 eV) has a higher *S* value than BPA (375.3283 eV) and MCN (317.70344 eV). These results were aligned with the calculated HOMO–LUMO gap, where the photocatalysts with the smallest HOMO–LUMO gap have the highest *η*, meaning the compound tends to donate electrons to an acceptor during the reaction. The maximum charge transfers (Δ*N*_max_) of BPA (1.9728 eV), MCN (2.3306 eV), and O-MCN (1.7180 eV) were calculated using eqn (14) to validate the previous results.

Based on the data obtained and eqn (15), BPA undergoes nucleophilic and electrophilic attacks when interacting with the MCN and O-MCN, respectively. To verify the result above, the electrophilic charge transfer (ECT) was determined using eqn (16) and (17). Based on the results, the ECT value of the BPA–O-MCN (−0.3578 eV) and BPA–MCN (0.2548 eV) complexes are greater and less than 0, respectively. Based on this result, the O-MCN and MCN perform nucleophilic and electrophilic substitution by donating or accepting an electron from BPA to form a non-covalent interaction.

14 Δ*N* = −*μ*^2^/*η*

15 ECT = (Δ*N*_max(1)_) − (Δ*N*_max(2)_)

16 *μ*_1_^2^/*η*_1_ − *μ*_2_^2^/*η*_2_ > 0

17 *μ*_1_^2^/*η*_1_ − *μ*_2_^2^/*η*_2_ < 0

Table 4 shows the quantum chemical descriptors of selected atoms involved in the formation of complexes between the catalysts (O-MCN and MCN) and the reactant (BPA). The Fukui function (*f*_*k*_^−^ and *f*_*k*_^+^), local softness (*σf*_*k*_^−^), and local electrophilicity indices (*ωf*_*k*_^+^) are local descriptors that represent a favoured region for the mechanistic insight into the photocatalytic reaction between the photocatalysts and the BPA.^68^ Chemists have used these approaches to identify which atom tends to donate or accept electrons. This study has two possible reaction mechanisms, O-MCN with BPA and MCN with BPA. As mentioned in the global reactivity section, O-MCN and BPA are prone to act as nucleophile and electrophile, respectively (first reaction). Meanwhile, the BPA and MCN are prone to act as the nucleophile and electrophile, respectively. Based on the Fukui function and local electrophilicity index (*f*_*k*_^+^ and *ωf*_*k*_^+^), the tendency of the atoms in the regions that undergo the nucleophilic attack in the MCN–BPA and O-MCN–BPA complexes can be arranged in descending order, H11 > H12 > H14 and 28H > 29H > 30H, respectively. For the electrophilic attack region (Fukui function: *f*_*k*_^−^, and local softness: *σf*_*k*_^−^) in the MCN–BPA and O-MCN–BPA complexes, the tendency of the atoms to act as the nucleophile are arranged in ascending order; 17C < 2O < 1O and 15N < 27N < 14N, respectively. However, the results of these approaches need to align with the hard and soft acid and base (HSAB) concept, where the hard and soft base atoms prefer to coordinate with the hard and soft acid atoms, respectively.^69^

**Table 4:** Calculated quantum chemical descriptors for the selected nucleophilic (*f*_*k*_^+^ and *ωf*_*k*_^+^) and electrophilic (*f*_*k*_^−^ and *σf*_*k*_^−^) attacks regions for the photocatalysts (O-MCN and MCN) and reactant (BPA)

Complex	Function	Atoms	*f*_*k*_^−^	*f*_*k*_^+^	*σf*_*k*_^−^	*ωf*_*k*_^+^
BPA–MCN	Electrophilic attack region (BPA)	1O	5.5810		76.9799	
		2O	5.7062		78.7064	
		17C	3.2001		44.1386	
BPA–MCN	Nucleophilic attack region (MCN)	11H		6.1797		0.0122
		12H		6.1770		0.0123
		14H		6.1742		0.0122
BPA–OMCN	Electrophilic attack region (OMCN)	14N	13.3499		211.4497	
		27N	12.7784		202.3986	
		15N	7.7199		122.2753	
BPA–OMCN	Nucleophilic attack region (BPA)	28H		4.8191		0.0105
		29H		4.8082		0.0104
		30H		4.6749		0.0102

## Conclusions

Mesoporosity and oxygen functionalities were successfully introduced in the framework of carbon nitrides through one-step thermal polymerization of urea, glucose utilizing nanodisc silica (NDS) from rice husk ash as a hard template. The oxygen doped MCN (O-MCN; 40 mg L^−1^) is active in the photodegradation of BPA under the visible light irradiation. Within 180 min, 97% of BPA (10 mg L^−1^) was successfully removed at pH 10. The higher porosity of O-MCN and sub-gap impurity states act as the trapping centre of the photo-induced charge carrier and inhibit the recombination of the electron–hole pairs. Scavenging test indicate that the major driving force of the photodegradation were h^+^ and O_2_˙^−^ rather than ˙OH. The intermediate identified from LC/TOF/MS analysis suggested that the BPA were photodegraded through hydroxylation, β-scission and oxidation. Density of states (DOS) analysis indicated that oxygen doping resulted a higher photoactivity value due to stronger localization and delocalization of the HOMO and LUMO in O-MCN than in MCN. Based on the DFT calculation, 14N (O-MCN), 1O (BPA) and 30H (BPA), 11H (MCN) were successfully identified as the reactive atom that undergo the nucleophilic and electrophilic attack during the formation of MCN-BPA and O-MCN–BPA complex, respectively. The precursors and synthesis method used in to synthesized O-MCN are greener and less tedious. To the best of our knowledge, not many research groups has used NDS from rice husk ash as a template for the synthesis of mesoporous carbon nitride. In addition of being able to remove higher concentration of BPA under visible light, the doping of oxygen using this method is able to preserve the surface area and created broader pore size distribution for photodegradation to take place effectively. Hence, these are the advantages of O-MCN.

## Conflicts of interest

The authors declare that they have no known competing financial interests or personal relationships that could have appeared to influence the work reported in this paper.

## Supplementary Material

RA-012-D2RA00995A-s001
