# Supplementary material for: Insight into the photodegradation mechanism of bisphenol-A by oxygen doped mesoporous carbon nitride under visible light irradiation and DFT calculations
Source: RSC Adv. 2022 Apr 6;12(17):10409–23. doi: 10.1039/d2ra00995a (PMC8984687; doi:10.1039/d2ra00995a)
Supplement: RA-012-D2RA00995A-s001 [file RA-012-D2RA00995A-s001.pdf]

Electronic Supplementary Material (ESI) for RSC Advances.
This journal is © The Royal Society of Chemistry 2022

AN INSIGHT INTO THE PHOTODEGRADATION MECHANISM OF BISPHENOL-A BY OXYGEN DOPED MESOPOROUS CARBON NITRIDE UNDER VISIBLE IRRADIATION AND DFT CALCULATION

Shittu Fatimah Bukola, Anwar Iqbal, Mohammad Norazmi Ahmad, Muhammad Rahimi Yusop, Mohamad Nasir Mohamad Ibrahim, Sumiyyah Sabar, Lee D. Wilson, Dede Heri Yuli Yanto

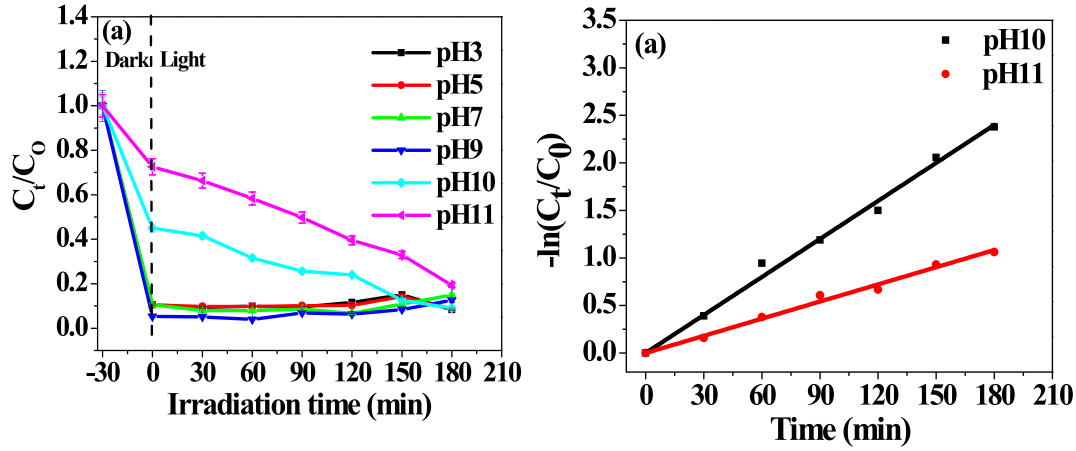

Fig. S1 The plot of (a) effect of initial pH of BPA and (b) pseudo-first-order kinetics of BPA photodegradation by O-MCN nanocomposite in different pH media on the removal of BPA ([BPA] = 20 mg L^{-1}; [O-MCN] = 50 mg L^{-1}).

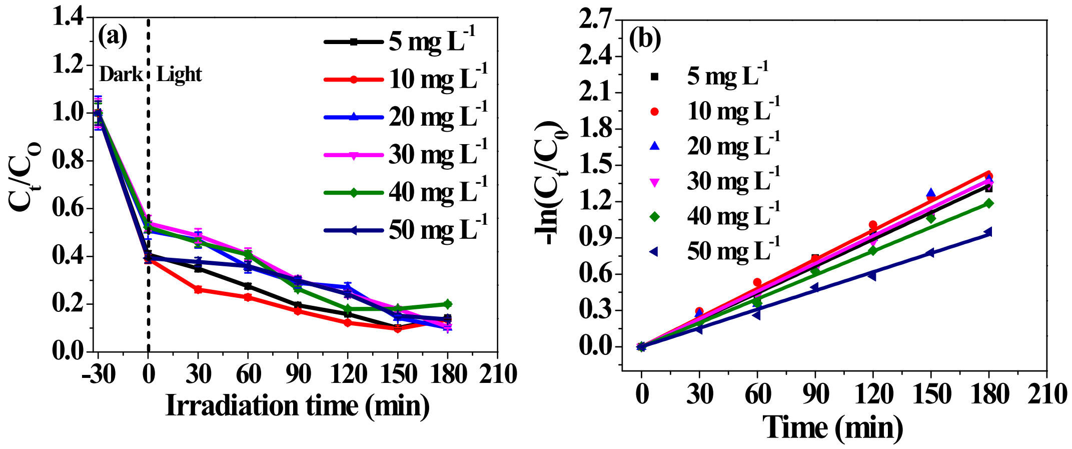

Fig. S2 The plot of (a) effect of initial concentration of BPA and (b) pseudo-first order kinetics of BPA photodegradation by O-MCN nanocomposite under different initial concentration of BPA solution. ([BPA] = 5, 10, 20, 30 40 and 50 mg L^{-1}; [0.2O-MCN] = 50 mg L^{-1}; pH = 10).

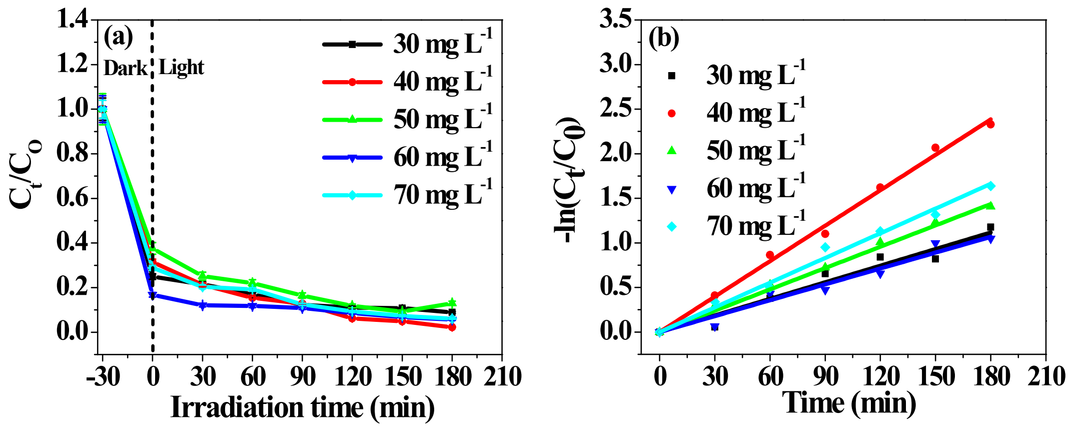

Fig. S3 The plot of (a) effect of catalyst dosage on BPA and (b) pseudo-first order kinetics of BPA photodegradation using different dosages of O-MCN nanocomposite. ([BPA] = 10 mg L^{-1}; [O-MCN] = 30, 40, 50, 60 and 70 mg L^{-1}; pH= 10.

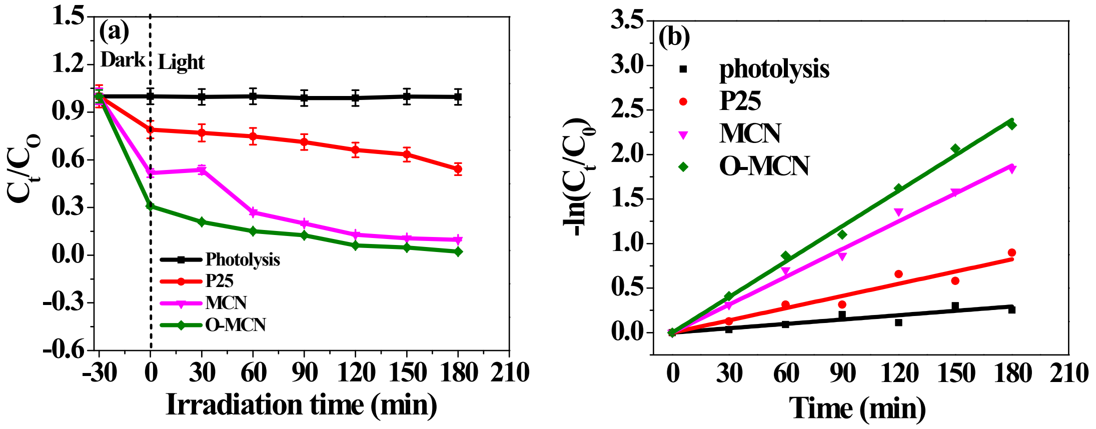

Fig. S4 The plot of (a) the effect of different synthesized photocatalyst and (b) pseudo-first order kinetics of BPA photodegradation using different synthesized against P25 on degradation of BPA ([BPA] = 10 mg L^{-1}; [catalyst] = 40 mg L^{-1}; pH= 10).
